# Stepping into emotions: investigating the effect of angry and fearful faces on forward stepping and quiet stance

**DOI:** 10.3389/fnhum.2024.1411246

**Published:** 2024-08-09

**Authors:** Angélique Lebert, Oscar Vilarroya, John Stins

**Affiliations:** ^1^Unitat de Recerca en Neurociència Cognitiva, Departament de Psiquiatria i Medicina Legal, Universitat Autònoma de Barcelona, Barcelona, Spain; ^2^Hospital del Mar Research Institute, Barcelona, Spain; ^3^Department of Human Movement Sciences, Faculty of Behavioural and Movement Sciences, Vrije Universiteit Amsterdam, Amsterdam Movement Sciences, Amsterdam, Netherlands

**Keywords:** emotion, faces, gaze direction, gait initiation, quiet stance, posture, approach-avoidance motivation

## Abstract

**Introduction:**

Facial expressions conveying an emotion may affect social interactions, such as approach- or avoidance-related behaviors. A specific facial feature is the gaze direction. An emotional facial expression such as anger will elicit distinct behavioral tendencies, depending on whether the angry gaze is directed toward the onlooker, or in a different direction. We tested whether facial expressions of anger and fear, combined with direct or averted gaze, elicit approach- or avoidance tendencies, using a go/no-go variant of the whole-body stepping task.

**Method:**

Healthy adults stood on a force plate, recording the center of pressure (COP). Participants were presented with angry or fearful faces; either with direct or averted gaze. Participants had to identify the emotion, and “depending on instructions- either make a single step forward, or remain in a quiet stance. From the COP of the forward steps, we derived parameters such as reaction time and step size. From the quiet standing trials we derived parameters of postural sway, indicative of postural “freeze.” We used analysis of variance to analyze the outcomes.

**Results and discussion:**

First, we found that steps were initiated faster with angry faces than with fearful faces, in line with existing literature. Second, we did not observe a significant effect of gaze direction. Forward steps with direct and averted gaze had similar COP characteristics. Finally, we had expected to find freeze (postural immobility) with fearful faces, but this was also not observed. We discuss various explanations for the finding, and implications for research into the motoric grounding of social interactions.

## 1 Introduction

The ability to produce and recognize emotional facial expressions has a long evolutionary history, characterized by the contraction of specific facial muscles and their adaptive function in both humans and animals (Waller and Micheletta, 2013). Over time, these basic facial responses have evolved to be used in more complex nonverbal communication processes, such as interpreting the intentions and goals of others (Erickson and Schulkin, 2003). Emotional processing likely serves a beneficial evolutionary purpose by allowing an organism to quickly adapt to a changing environment (Frijda, 2010; Lang and Bradley, 2010; Roelofs, 2017). For example, an angry facial expression may involve displaying a set of sharp teeth. This may signal potential threat, and this induces an avoidance reaction in the observer, thereby motivating them to move away from danger. This triggering of goal-directed behavior in response to emotional faces suggests that perception and action have co-evolved and are thus tightly linked.

This connection can be understood in terms of behavioral affordances related to visual perception, which refer to opportunities for interaction or response to a visual stimulus (Gibson, 2014). Adams et al. (2017) propose that the response to facial perception can be understood in terms of behavioral affordances. These affordances are presumed to be innate but can be influenced by individuals' adjustments to their environment, which are related to their sensitivity to stimulus features and can vary significantly within an individual (i.e., personality) and across situations (i.e., context). In real life, emotions can occur simultaneously, for instance when facing a threatening situation one may experience both fear, anger and disgust at the same time (Dailey et al., 2002). Furthermore, the importance of emotions in shaping human behavior and decision-making processes is well-established in the literature (de Gelder et al., 2006; Botta et al., 2022). Emotions play a crucial role in decision-making by triggering adaptive behavioral responses that have evolved through evolution to help individuals navigate complex and ever-changing environments social. These responses guide individuals to pursue appetitive goals while steering clear of potential threats (Frijda, 1986; Frijda et al., 1989; Lang, 1995; Lang et al., 2013). Numerous researchers have delved into the influence of emotional stimuli on approach- or avoidance related motor responses, particularly examining their impact on directional arm movements, which has revealed varying effects across different emotional states. For instance, Wilkowski and Meier (2010) observed that angry expressions tend to facilitate approach behaviors. More specifically they showed that participants consistently exhibited faster initiation of approach movements toward angry facial expressions, compared to neutral or fearful expressions, particularly when physical approach was perceived as an effective strategy for addressing the social challenge posed by angry facial expressions.

Within this line of research, a recent series of studies has shed light on a key insight: emotional facial expressions elicit consistent and replicable behavioral responses exclusively when they align with participants' specific objectives, indicating a context-dependent nature of emotional effects (Mirabella, 2018; Mancini et al., 2020, 2022; Calbi et al., 2022). Illustrating this point, Mirabella (2018), using two versions of a Go/No-go task, showed that emotional facial expressions (happy and fearful) affect motor readiness and accuracy of reaching arm movements only when the stimuli are task-relevant. More specifically, when participants focused on emotional content, reaction time and omission errors were higher for fearful faces compared to happy stimuli. In contrast, there was no difference when participants were instructed to react to a neutral stimulus component, in this case gender. Such results have been replicated and extended by including angry faces (Mancini et al., 2020). Mancini et al. (2022) and Calbi et al. (2022) showed that inhibitory control, a key executive function, is impacted by facial or body posture emotions respectively, but only when they are task-relevant. The authors showed that the percentage of commission errors (i.e., instances in which participants moved although they had not) were higher for happy faces than for fearful faces.

Additionally, as proposed by Said et al. (2011), emotional facial expressions are not processed independently of contextual cues provided by the face. Other facial cues such as the gaze direction of another person may provide additional assistance in identifying emotional facial expressions (Adams and Kleck, 2003). Simply put, it matters a great deal if an angry expression is directed toward me, or toward my neighbor. According to the shared signal hypothesis (SSH) proposed by Adams and Kleck (2005), emotion processing is enhanced when the gaze direction corresponds to the motivational orientation of the expressed emotion. For example, expressions such as happiness or anger would be perceived more quickly and intensely by the observer when associated with direct gaze (sharing an approach motivation; Adams and Kleck, 2003, 2005; Willis et al., 2011; Pönkänen and Hietanen, 2012). Indeed, an angry face combined with a direct gaze might be more threatening to the observer than an averted gaze, as the direct gaze would designate the observer as the target of the threat. Conversely, an averted gaze coupled with a fearful face may signal a danger in the immediate environment, potentially hazardous to the observer (Adams and Franklin, 2009; Adams et al., 2017). The ability to detect the source of a threat requires accurate interpretation of emotional facial expressions itself and the direction of gaze, as these two signals interrelate. In social interactions, both emotion and gaze direction allow observers to simultaneously assess others' intentions and identify external cues that may be favorable or unfavorable, or even represent a threat. Consistent with previous findings, several authors observed that the perception of gaze direction was influenced by the emotional faces; participants were more likely to perceive angry faces as looking directly at them, while fearful faces were perceived as looking more toward the environment (Ewbank et al., 2009; Jun et al., 2013; Lebert et al., 2021).

In summary, based on the SSH, happy and angry faces would promote approach behaviors, while fearful, sad and disgusted faces would promote avoidance behaviors (Adams and Kleck, 2003, 2005; Sander et al., 2007; Willis et al., 2011; Pönkänen and Hietanen, 2012). A more recent, and potentially more ecological way of assessing the relationship between emotional stimuli and motivated behavior (i.e., approach or avoid) involves the measurement of whole-body movements. This method considers the fact that moving the entire body toward (approach) or away from (avoid) an emotional stimulus alters the physical distance between the individual and the stimulus. It is worth noting, however, that the effects on behaviors are not always clear-cut, and there are discrepancies in the reported results. Several authors have shown that pleasant stimuli lead to faster initiation of step forward compared to unpleasant stimuli (Gélat et al., 2011; Stins et al., 2011a; Yiou et al., 2014). Also, no effect of viewing unpleasant stimuli on organization of backward steps has been reported in the literature (Stins et al., 2011a; Yiou et al., 2014; Bouman and Stins, 2018). This lack of effect may be explained by the fact that in everyday life, escaping danger does not involve taking a backward step, which arguably leads to slower and less stable movements compared to the more “natural” forward step. Finally, Mirabella et al. (2022) demonstrated that emotional facial expressions significantly influence gait parameters only when participants are explicitly instructed to move according to the valence of the picture, facilitating an approach toward pleasant faces compared to unpleasant ones. To our knowledge, only Mirabella et al. (2022) performed a whole-body go no/go task, involving emotional items. In that study, only the go trials were analyzed, using an optoelectronic movement registration system. However, it can be argued that also the no/go trials are informative, because during quiet standing also effects of emotion can be demonstrated, such as postural “freeze” and spontaneous backward body displacements, when faced with a threat (Hillman et al., 2004; Azevedo et al., 2005; Facchinetti et al., 2006; Roelofs et al., 2010; Stins and Beek, 2011). In this regard, an insightful indicator for assessing the influence of an impending threat could be freezing behavior—a state in which the body undergoes physiological and somatic preparations for action when the threat is distant and fear levels are relatively low (Blanchard et al., 1986; Roelofs et al., 2010; Borgomaneri et al., 2015; Botta et al., 2022). During freezing, the body is temporarily immobile, which would be manifest as very little postural sway. Conversely, arousing items could lead to an increase in sway. In this regard, the study of Bouman and Stins (2018) (described above) observed an effect on the postural sway preceding the backward step, specifically an increase in postural sway for high arousal stimuli, regardless of valence.

The objective of this study is to delve into the intricate interplay between social emotional cues and postural behavior. More specifically, we aim to explore how specific emotions (anger and fear) combined with different gaze directions (direct and deviated) influence posture. While both emotions share an unpleasant valence, they have distinct motivational properties – anger promotes approach behaviors (Carver and Harmon-Jones, 2009) while fear triggers freezing and avoidance behaviors. To this end, we used a force plate that allows recording of the body center of pressure, both during go trials (forward stepping) and no/go trials (quiet stance) in response to emotional faces of anger or fear, combined with either a direct or deviated gaze. Regarding the planning and organization of a forward step, we predict that the reaction times will be shorter in response to angry faces relative to fearful faces. Moreover, this effect will be increased for potentially threatening conditions to the observer (angry faces with direct gaze and fearful faces with deviated gaze) compared to less threatening conditions (angry faces with deviated gaze and fearful faces with direct gaze). Concerning static posture, we hypothesize to observe a stronger freezing behavior (i.e., greater reduction in sway) in response to fearful faces relative to angry faces, and even more so for conditions that are potentially threatening to the observer due to the gaze direction.

## 2 Method

### 2.1 Participants

Based on comparable studies investigating the effect of emotion on posture (Stins et al., 2011b; Eerland et al., 2012; Fawver et al., 2015) a sample of 22 adults (nine males; 13 females; mean age 26 y; sd 4.5 y) completed the study and was deemed sufficient. No power analysis was performed beforehand. Participants had normal or corrected-to-normal vision and had no neurological disorders or injuries that prevented them from participating. Twenty-one participants had a self-declared right-foot preference to kick a ball; one participant had a self-declared left-foot preference. All participants signed an informed consent form. The study was approved by the local ethical committee of the Vrije Universiteit, Amsterdam.

### 2.2 Stimuli

**Figure 1:**
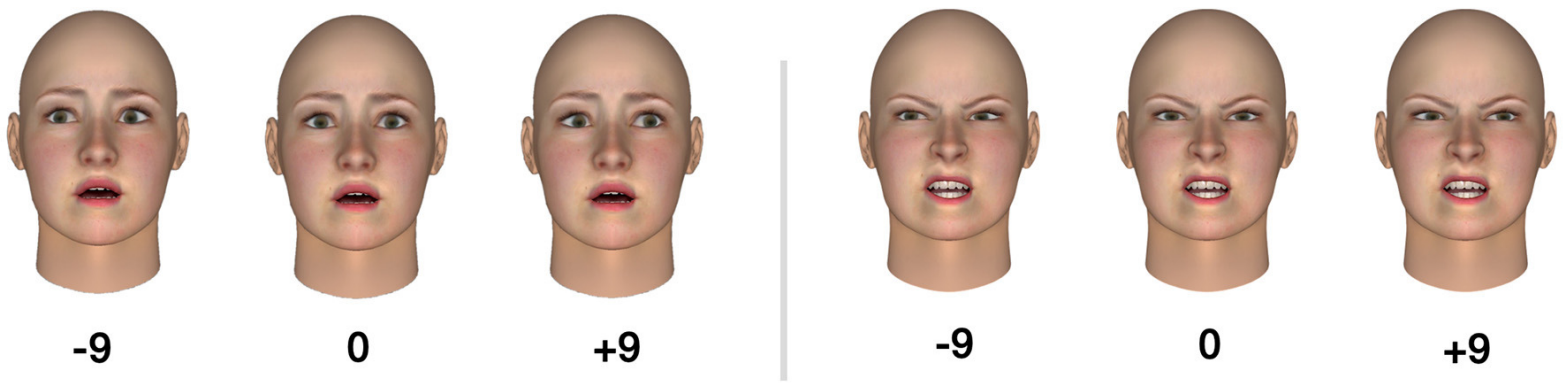
Example stimuli of three fearful faces **(left)** and three angry faces **(right)**. The center face is a direct gaze, facing the observer. The center faces are flanked by faces with averted gaze; 9° leftward and 9° rightward.

Stimuli consisted of a set of Caucasian faces generated using FaceGen Modeler software. Hair and other markings such as jewelry or tattoos were absent so that only the central face area was visible. There were two identities for both genders (M/F), displaying either an angry or fearful emotion. Importantly, in some cases the eye gaze was directly forward (i.e., facing the viewer), or deviated to the left, or deviated to the right. In the deviated gaze condition, the iris was shifted by nine pixels to the left or to the right, so that there is no ambiguity about the deviated direction of gaze. This resulted in a set of 24 unique facial stimuli: 2 (emotions: angry vs. fearful) * 2 (genders: female vs. male) * 2 (identities: first vs. second identity) * 3 (gaze directions: direct gaze vs shifted to the right vs to the left). Each stimulus was repeated twice (instructions: go vs. no-go trials). Example face stimuli are shown in Figure 1. Similar faces (but expressing a broader range of emotions) were used in previous studies where a pre-test was initially conducted with an independent sample of fifty participants (Lebert et al., 2021, 2024). We ensured that participants had correctly identified the displayed emotions according to their instructed motor responses. In the study employing these faces for the first time (Lebert et al., 2021), the visual angles of the face measured 10.57° (height) x 8.01° (width), with a 9-pixel deviation (i.e., deviated gaze) corresponding to a visual angle of 0.139°; whereas in our current study, the visual angles of the face measured 16.37° x 12.47°, with a 9-pixel deviation corresponding to a visual angle of 0.217°.

### 2.3 Apparatus

Postural sway data were collected using a custom-made 1 x 1 m force plate, measuring forces in the x-, y-, and z-direction. From the forces we calculated the COP time series. Stimuli were shown at eye-level on a 55“ monitor, placed ~1.5 m from the starting position of the participant. In order to synchronize the onset of the visual stimuli with the continuous force plate measurement, we used a small light sensor which was attached to the bottom left corner of the screen. Together with each visual stimulus, a small white square (not visible to the participant) was presented. At stimulus offset the white square disappeared again. This resulted in a square wave pattern timed exactly with onset and offset of each stimulus. Data of the light sensor was sampled simultaneously with the force plate channels, enabling offline identification of stimulus events.

Initially, we adopted a very high sampling frequency of 1,000 Hz for the sole purpose of accurately identifying the onset of the white square (due to the monitor's inevitable flickering). However, during the course of the experiment the measurement PC sometimes exhibited memory problems, resulting in a premature abortion of data collection (see below). Consequently, we later adjusted the sampling frequency to 500 Hz. In the actual data analysis, we downsampled all data to 100 Hz, as postural frequencies exceeding this value are unlikely to have a biomechanical origin and instead reflect noise.

### 2.4 Procedure

We implemented a whole-body version of the go/no-go task. Participants were all positioned slightly at the rear of the force plate and were instructed to execute a single forward step as fast and accurately as possible upon the appearance of a “go” signal, while remaining motionless on the force plate when the “no-go” signal was presented. In other words, performance of the go/no-go task required accurate identification of the emotional expression (regardless of gaze direction). Participants were instructed to initiate all steps with their right leg. In one block of trials the go signal was the angry face while the no-go signal corresponded to the fearful face. Subsequently, after the block of trials was completed, the instruction was reversed, and the same 24 facial stimuli were presented again. Stimulus presentation was randomized and instructions order was counterbalanced between participants. We thus have a complete within design where all stimuli and all stimulus-response assignments are present. Preceding each trial block, participants underwent five practice trials.

**Figure 2:**
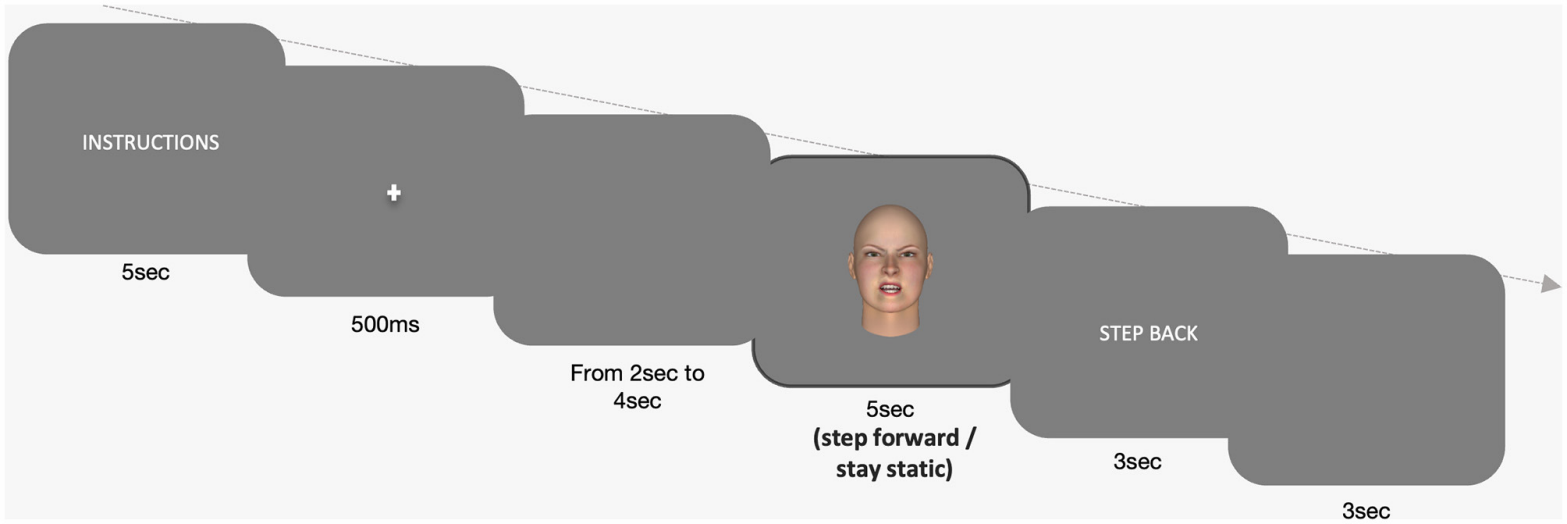
Timeline of experimental events, including instruction display (5 s), fixation cross (500 ms), black screen (random 2–4 s), face stimulus presentation (5 s), participant responses to go and no-go stimuli, and instruction to “step back” (displayed for 3 s) before a final black screen (3 s).

The timing of events was as follows (see Figure 2): (1) a screen containing the instructions, shown prior to each trial. The instructions were displayed either in Dutch or in English (dependent on the preferred language of the participant) and remained on the screen for 5 s. (2) A 500 ms small white fixation cross in the center of the screen. (3) A black screen, lasting a random 2–4 s. (4) Presentation of the face stimulus for 5 s. In the case of a go stimulus, participants had to take a forward step and to remain still in the new (anterior) position of the force plate. In the case of a no-go stimulus participants had to maintain a quiet stance. (5) The monitor displayed the message “step back” for 3 s. (6) Finally a black screen for another 3 s. The entire experiment lasted about 40 min. Prior to the experiment, participants received written and verbal instructions. The required movement pattern (i.e., the step) was demonstrated by the experimenter.

### 2.5 Data analysis

Prior to data analysis we had to remove a total of five errors; four involving failing to step with a go trial, and one involving stepping with a no-go trial. This demonstrates that the emotions were generally easily identifiable.

**Figure 3:**
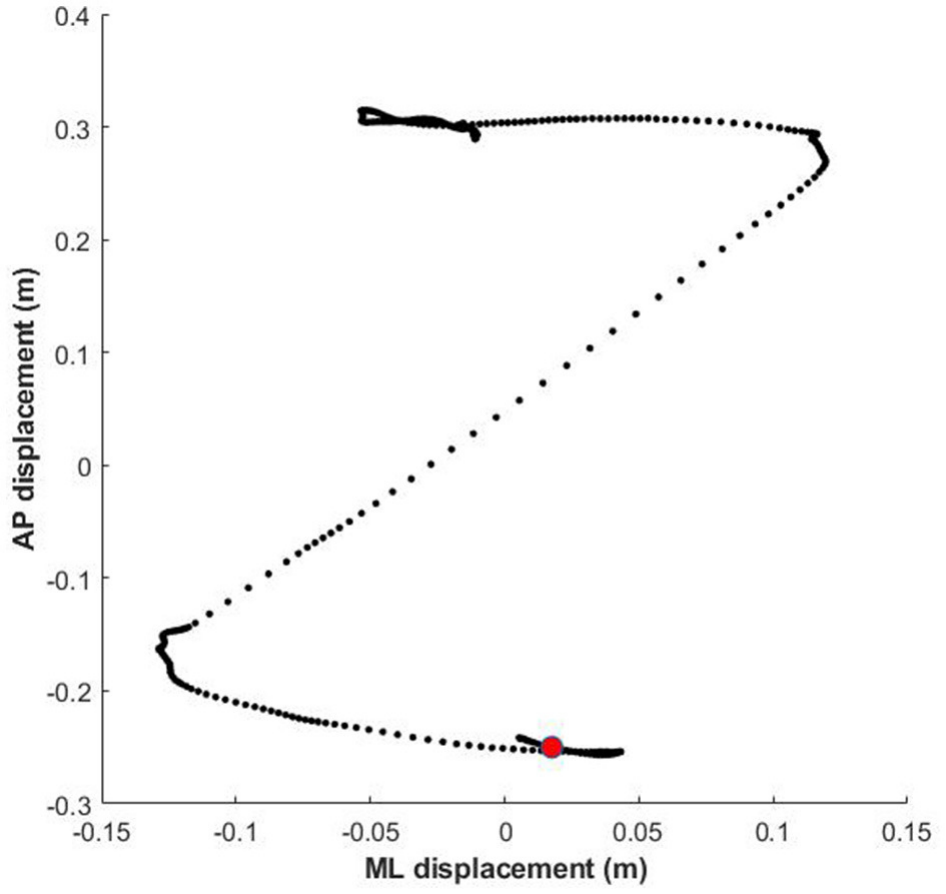
Example COP profile of a forward step (angry face; averted gaze). Each dot represents one sample (time interval 10 ms). The red circle denotes the reaction time for this particular step, i.e., the first discernible weight shift from quiet standing. The initial leftward displacement of the COP represents the unloading of the **(right)** swing leg and the loading of the left stance leg. This is followed by the forward acceleration, powered by the left foot. In this example the RT was 430 ms, and the step size 55 cm.

COP time series were downsampled to 100 Hz and smoothed with a 5-point moving average. We used custom made Matlab scripts to identify the peaks in the light sensor signal, to identify the corresponding COP segments and to extract relevant postural parameters. For the go trials we analyzed a number of classical GI parameters: (1) reaction time, i.e., the time interval between stimulus onset and movement initiation. Following previous studies (e.g., Bouman et al., 2021), we identified movement onset as the moment in time at which the force in the anterior direction exceeded 5 N. (2) step size; the difference between the initial standing position and the final position. (3) APA; the distance along the APA axis between the initial COP position and the most posterior position, which for biomechanical reasons is related to the build up of speed in the forward direction (see Figure 3).

For the no-go trials we analyzed several parameters indicative of quiet standing performance: the standard deviation of the COP time series in the AP and ML direction, and the sway path length. We performed analyzes of variance on the postural outcome measures, separately for the go and the no go trials. The factors were emotion (angry/fearful) and gaze (direct or averted, i.e., we collapsed over left and right gaze deviation).

## 3 Results

Due to technical errors the data of one (female) participant had to be discarded. In addition, due to occasional premature termination of the data collection, 15 trials were lost.

### 3.1 Gait initiation

**Figure 4:**
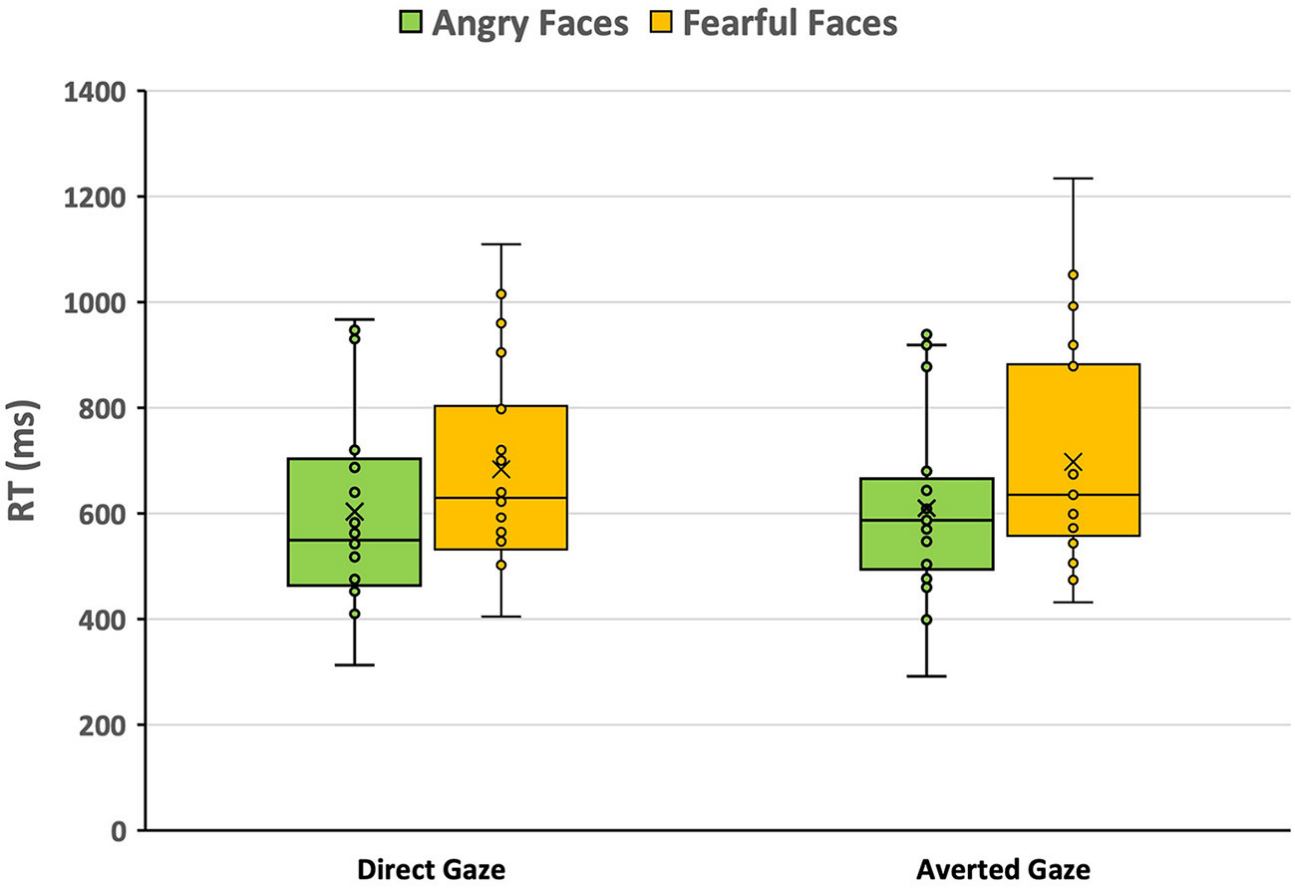
Box plots of reaction times (in ms) in response to emotional faces (angry vs. fearful) associated with either a direct or an averted gaze. Each dot represents the mean of a single participant; the “x” represents the condition mean, and the horizontal line is the median.

Prior to analysis we removed steps that were initiated too soon (< 200 ms) or too late (> 1,500 ms). This resulted in removal of 30 trials, but it never resulted in empty cells. Analysis of the RTs only revealed a main effect of emotion; *F*_(1, 20) = 12.21, *p* = 0.002; $\eta_{p}^{2}$ = 0.38. This effect was due to the considerably faster step initiation with angry faces (607 ms) compared to fearful faces (691 ms; see Figure 4). The effect of gaze was not significant; *F*_(1, 20) = 0.73, *p* = 0.404; $\eta_{p}^{2}$ = 0.03, nor was the interaction of emotion and gaze; *F*_(1, 20) = 0.67, *p* = 0.79; $\eta_{p}^{2}$ = 0.00. Analysis of APA and of step size (with the same trials removed) yielded no significant main or interaction effects. The average step size was about 40 cm, ranging between 20 and 60 cm.

### 3.2 Quiet standing

Analysis of quiet standing yielded no significant main or interaction effects. The average standard deviation of sway in the AP direction was 3.2 mm; in the ML direction 3.5 mm. The average sway path length was 89.5 mm.

## 4 Discussion

In this study, we aimed to investigate the effect of angry and fearful faces on the control of forward stepping and quiet stance. In our predictions, we posited that anger and fear have distinct motivational properties of approach and avoidance, respectively, and that the combination of these emotions with gaze direction would further enhance these motivational tendencies, thus differentially affecting motor correlates (Adams and Kleck, 2005). Using a go/no-go paradigm, we measured both gait initiation and quiet standing parameters in response to emotional faces with either a direct or deviated gaze.

First, we predicted that gait initiation would be faster in response to angry faces compared to fearful faces, which is indeed what we found. This finding aligns with previous research showing that angry expressions tend to facilitate approach behaviors (Wilkowski and Meier, 2010; Mancini et al., 2020). This shorter reaction times when initiating forward steps in response to angry faces could reflect a faster motor readiness to approach potentially challenging social situations. It is essential to note that the distinction between fear and anger in terms of their impact on motor responses is not clear-cut in the existing literature. While fear and anger are often considered interchangeable negative emotions, our study highlights that they convey distinct social signals and, consequently, modulate actions differently. Specifically, angry faces, in contrast to fearful ones, are associated with a direct threat in the form of aggression toward the observer, thus eliciting an immediate need for action (Adams and Franklin, 2009; Adams et al., 2017).

We initially hypothesized that the combination of gaze direction and emotional facial expression would have a differential effect on forward stepping. Contrary to our expectations, we did not observe a significant impact of gaze direction on GI. As an explanation of this unexpected finding, one hypothesis could be that emotional facial expressions would have a more dominant influence on approach behavior than gaze direction. Especially in threatening situations, where efficient detection of danger is crucial, it appears that emotional expressions may determine motor responses more than gaze direction. We have no explanation for this finding, but it could be that gaze direction (which after all was task irrelevant in this study) does not affect performance, since the task was to identify the emotion regardless of gaze, and execute the assigned motor response. Note that in previous studies, gaze direction was a task-relevant parameter and did seem to affect emotional responses (Ewbank et al., 2009; Jun et al., 2013; Lebert et al., 2021) and postural control (Lebert et al., 2021).

Lastly, we focused on the impact of postural sway during no-go trials. We hypothesized that participants would exhibit stronger freezing behavior, characterized by a greater reduction in sway, in response to fearful faces compared to angry faces, especially in potentially threatening conditions (Azevedo et al., 2005; Facchinetti et al., 2006; Roelofs et al., 2010; Stins and Beek, 2011). However, this prediction was not confirmed, as we did not observe significant differences in postural variability between emotional conditions during quiet standing. Based on these results, we conclude that the freeze response, typically associated with threat detection in the initial moments, may not have been elicited to a significant extent. A potential reason could be that the experiment consisted of a mixture of quiet standing trials and gait initiation trials. Traditional studies on the freeze response only involve quiet standing recordings. This novel go /no-go paradigm may not be suitable to elicit a freezing response. The go / no-go paradigm is often used to measure sustained attention over a long period of time, whereas our paradigm aims to unravel spontaneous postural adjustments in response to emotional faces with either a direct or averted gaze.

In future studies a wider range of emotions should be employed. For present purposes we focused on two emotions (fear and anger) with differing motivational properties, but other emotions such as happiness and disgust, combined with various gaze angles, can be used to obtain more insight in how, and to what extent, there prime the motor system. Another limitation is that we did not collect data on personality type. The results of Lebert et al. (2020) suggest that postural effects are modulated by personality.

To our knowledge, this is the first study implementing a go/no-go paradigm to investigate the influence of emotional expressions combined with direct or deviated gaze on both forward stepping and quiet standing. Our findings suggest that facial cues do not carry equal weight in our social interactions and, more broadly, in perception-action links. Exploring how emotions and gaze direction modulate these responses in contexts more closely resembling real-life social situations can offer valuable insights into comprehending human social interactions.

## Data availability statement

The raw data supporting the conclusions of this article will be made available by the authors, without undue reservation.

## Ethics statement

The studies involving humans were approved by Vaste Commissie Wetenschap en Ethiek (The Scientific and Ethical Review Board; VCWE). The studies were conducted in accordance with the local legislation and institutional requirements. The participants provided their written informed consent to participate in this study.

## Author contributions

AL: Conceptualization, Formal analysis, Investigation, Methodology, Writing – original draft, Writing – review & editing. OV: Writing – review & editing. JS: Conceptualization, Data curation, Formal analysis, Methodology, Software, Supervision, Writing – original draft, Writing – review & editing.
